# Differential roles of miR‐15a/16‐1 and miR‐497/195 clusters in immune cell development and homeostasis

**DOI:** 10.1111/febs.15493

**Published:** 2020-08-04

**Authors:** Katharina Hutter, Thomas Rülicke, Mathias Drach, Lill Andersen, Andreas Villunger, Sebastian Herzog

**Affiliations:** ^1^ Institute of Developmental Immunology Biocenter Medical University Innsbruck Innsbruck Austria; ^2^ Institute of Laboratory Animal Science University of Veterinary Medicine Vienna Vienna Austria; ^3^ Department of Dermatology, Venereology and Allergology Cantonal Hospital St. Gallen St. Gallen Switzerland; ^4^ CeMM Research Center for Molecular Medicine of the Austrian Academy of Sciences Vienna Austria; ^5^ Ludwig Boltzmann Institute for Rare and Undiagnosed Diseases Vienna Austria

**Keywords:** hematopoiesis, leukemia, miR‐15, miR‐195, miR‐497

## Abstract

MicroRNAs (miRNAs) post‐transcriptionally repress almost all genes in mammals and thereby form an additional layer of gene regulation. As such, miRNAs impact on nearly every physiological process and have also been associated with cancer. Prominent examples of such miRNAs can be found in the miR‐15 family, composed of the bicistronic clusters miR‐15a/16‐1, miR‐15b/16‐2, and miR‐497/195. In particular, the miR‐15a/16‐1 cluster is deleted in almost two thirds of all chronic B lymphocytic leukemia (CLL) cases, a phenotype that is also recapitulated by miR‐15a/16‐1‐deficient as well as miR‐15b/16‐2‐deficient mice. Under physiological conditions, those two clusters have been implicated in T‐cell function, and B‐cell and natural killer (NK) cell development; however, it is unclear whether miR‐497 and miR‐195 confer similar roles in health and disease. Here, we have generated a conditional mouse model for tissue‐specific deletion of miR‐497 and miR‐195. While mice lacking miR‐15a/16‐1 in the hematopoietic compartment developed clear signs of CLL over time, aging mice deficient for miR‐497/195 did not show such a phenotype. Likewise, loss of miR‐15a/16‐1 impaired NK and early B‐cell development, whereas miR‐497/195 was dispensable for these processes. In fact, a detailed analysis of miR‐497/195‐deficient mice did not reveal any effect on steady‐state hematopoiesis or immune cell function. Unexpectedly, even whole‐body deletion of the cluster was well‐tolerated and had no obvious impact on embryonic development or healthy life span. Therefore, we postulate that the miR‐497/195 cluster is redundant to its paralog clusters or that its functional relevance is restricted to certain physiological and pathological conditions.

## Graphical abstract

The miR‐15a/16‐1 and miR‐15b/16‐2 clusters regulate hematopoiesis and are key tumor suppressors in chronic lymphocytic leukemia. However, little is known about the third cluster of the miR‐15 family, miR‐497/195. Here, we show that miR‐497/195 is dispensable for immune cell development and function, and that its loss does not provoke any overt malignancies. This suggests a redundant role for miR‐497/195 or that its functional relevance is restricted to certain conditions.

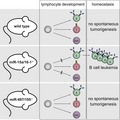

**Abbreviations**
Bcl2: B‐cell lymphoma 2
CLL: chronic lymphocytic leukemia
CRISPR: clustered regularly interspaced short palindromic repeats
ES: embryonic stem
Mcl1: myeloid cell leukemia 1
miRNA: microRNA
NK: natural killer
NP‐CGG: 4‐hydroxy‐3‐nitrophenylacetyl‐Chicken Gamma Globulin
sgRNA: single‐guide RNA
TNP: 2,4,6‐trinitrophenyl

## Introduction

MicroRNAs (miRNAs) are small noncoding RNAs that sequence‐specifically repress gene expression by binding to the 3′‐UTR of their respective target mRNAs [1]. Thus, miRNAs provide an additional layer of gene regulation, thereby affecting physiological as well as pathological processes. In cancer, miRNAs can exert both tumor‐promoting and tumor‐suppressive functions, depending on the miRNA and tumor type [2]. One prominent group of tumor‐suppressive miRNAs is the miR‐15 family, consisting of the three bicistronic clusters miR‐15a/16‐1, miR‐15b/16‐2, and miR‐497/195 [3]. In particular, the miR‐15a/16‐1 cluster is well known for its tumor‐suppressive function in chronic B lymphocytic leukemia (CLL), the most common adult leukemia in the Western world. CLL is characterized by the clonal expansion of CD5^+^ B cells in blood, bone marrow, and secondary lymphoid tissues, and appears to be driven by altered cellular signaling and typical genetic aberrations [4]. Intriguingly, about two thirds of all patients harbor a mutation or genomic deletion in the chromosomal region that encodes the miR‐15a/16‐1 cluster, suggesting a causal relationship between miR‐15a/16‐1 loss and leukemogenesis [5, 6, 7]. Indeed, a knockout mouse model lacking the miR‐15a/16‐1 cluster recapitulates the human disease and is characterized by the aberrant expansion of the CD5^+^ B‐cell pool typical for CLL [8]. Since all members of the miR‐15 family share the same seed sequence, and therefore regulate the same or at least overlapping target genes [1], it is not surprising that loss of the paralog miR‐15b/16‐2 cluster is also linked to CLL [9]. However, it remains unclear whether the third cluster, miR‐497/195, exerts a similar role.

Beyond CLL, a tumor‐suppressive function of the miR‐15 family has also been described for several other malignancies, which may be explained by its repressive effect on many proto‐oncogenes such as Bcl2, Mcl1, c‐Myb, and the cyclins D1, D3, and E3 [10, 11, 12]. The miR‐15b/16‐2 cluster, for example, has been described as a tumor suppressor in glioma and osteosarcoma, whereas the miR‐497/195 cluster appears to suppress tumor development in hepatocellular carcinoma, colorectal cancer, breast cancer, melanoma, and many other tumor types [13, 14, 15]. To our knowledge, however, this tumor‐suppressive potential has not been validated in miR‐497/195‐deficient mice.

In addition to its role in cancer, recent data begin to unravel physiological functions of the miR‐15 family, in particular miR‐15a/16‐1 and miR‐15b/16‐2. In early B‐cell development, for example, a knockdown of the miR‐15 family results in impaired differentiation and enhanced proliferation of pre‐B cells [11], suggesting that miR‐15 family members may preserve tissue homeostasis. Along the same line, the knockout of the miR‐15a/16‐1 cluster was reported to partially block natural killer (NK) cell maturation in the spleen [16]. In T cells, deletion of both the miR‐15a/16‐1 and miR‐15b/16‐2 clusters enhances proliferation and promotes memory cell formation upon lymphocytic choriomeningitis virus infection [17]. The miR‐497/195 cluster, on the other hand, appears to regulate angiogenesis and osteogenesis [18], but nothing is known about its physiological role in hematopoietic and other tissues.

To address whether the miR‐497/195 cluster is also implicated in CLL suppression, in immune cell development or immune function, we generated a conditional knockout mouse model. Comparing mice that lack miR‐15a/16‐1 or miR‐497/195 in the hematopoietic compartment upon Vav‐Cre‐mediated deletion confirmed reported roles for miR‐15a/16‐1 but failed to reveal overlapping roles in CLL control or in establishing hematopoiesis for miR‐497/195. Moreover, homeostasis and responsiveness of the immune system were not altered in tissue‐specific recombined miR‐497/195^fl/fl^ Vav‐Cre mice. Remarkably, even whole‐body deletion of the cluster was well‐tolerated and had no obvious impact on embryogenesis, tissue homeostasis, or healthy life span.

## Results

### Generation of miR‐497/195 conditional knockout mice

**Fig. 1:**
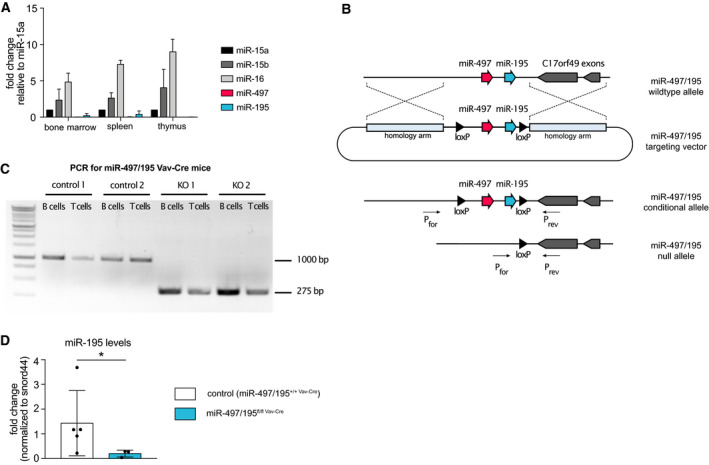
Generation of miR‐497/195 conditional knockout mice. (A) Expression levels of the different miR‐15 family members were analyzed by qPCR in indicated organs of C57BL/6N mice (*n* = 3). In individual organs, the levels of each miRNA were normalized to miR‐15a expression. (B) Targeting strategy for deletion of the miR‐497/195 cluster. The knock‐in allele was generated by CRISPR/Cas9‐induced targeting of murine ES cells with a DNA template containing the miR‐497/195 cluster flanked by loxP sites (black triangles) and two homology arms. Cre‐mediated deletion of the loxP‐flanked DNA region creates the miR‐497/195 null allele. The primer binding sites for genotyping are indicated as P_for_ and P_rev_. (C) Endpoint PCR on genomic DNA of FACS‐sorted B and T cells from two control (miR‐497/195^+/+ Vav‐Cre^) and two miR‐497/195^fl/fl Vav‐Cre^ mice. A fragment at 1000 bp shows the wild‐type allele, whereas a 275‐bp fragment indicates a deletion of the loxP‐flanked miR‐497/195 region. Data shown are representative of two independent experiments. (D) Quantitative PCR for the expression of mature miR‐195 in B cells derived from miR‐497/195^+/+ Vav‐Cre^ (*n* = 5) and miR‐497/195^fl/fl Vav‐Cre^ mice (*n* = 4). Both groups were statistically compared by an unpaired two‐tailed Student's *t*‐test. Error bars depict the standard deviation of the mean. *P < 0.05.

Given that all three paralog cluster genes of the miR‐15 family are located on different chromosomes and are embedded in distinct host genes, we first evaluated their expression levels in total bone marrow, spleen, and thymus (Fig. 1A). In analogy to previous reports [19, 20], we found comparable expression of miR‐15a/16‐1 and miR‐15b/16‐2 in all tissues, with miR‐497/195 being expressed at significantly lower levels. Most likely, this reflects the overall weak miR‐497/195 expression in immune cells that have been demonstrated by small RNA sequencing [19, 20]. However, we cannot rule out that distinct cell types found in these hematopoietic organs may also display higher miR‐497/195 levels.

To study potentially overlapping roles of miR‐497/195 with other members of the family in hematopoietic tissues, we generated a conditional miR‐497/195 knockout mouse model. In order to do so, we flanked the miR‐497/195 coding region with two loxP sites in mouse embryonic stem (ES) cells (Fig. 1B) using CRISPR‐based genome editing to avoid the need to introduce a selection marker, thereby preserving the overall genomic organization of the targeted allele (hereafter referred to as miR‐497/195^fl^). To investigate the role of the miR‐497/195 cluster in hematopoiesis and immunity, homozygous miR‐497/195^fl/fl^ mice were crossed onto the Vav‐Cre strain which efficiently deletes loxP‐flanked sequences already in hematopoietic stem cells and hence throughout the hematopoietic lineage [21] (Fig. 1B). Loss of the miR‐497/195 cluster was confirmed in both B and T cells of miR‐497/195^fl/fl Vav‐Cre^ mice by endpoint PCR (Fig. 1C). Amplification of a genomic DNA fragment of B and T cells isolated from control mice containing the miR‐497/195 cluster generated a 1000‐bp fragment, whereas deletion of the cluster in immune cells shortened the amplicon to about 275 bp. Consequently, miR‐195 was no longer expressed in sorted miR‐497/195^fl/fl Vav‐Cre^ B cells (Fig. 1D).

### Loss of miR‐15a/16‐1 but not of miR‐497/195 suffices to drive a CLL‐like phenotype

**Fig. 2:**
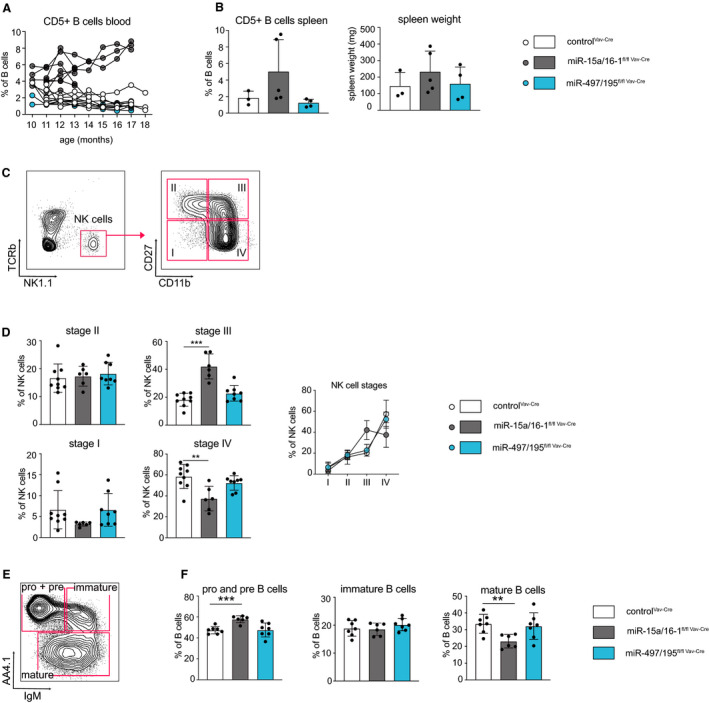
Loss of miR‐15a/16‐1 but not miR‐497/195 promotes a CLL‐like phenotype and impairs NK and early B‐cell development. (A) Blood of control, miR‐15a/16‐1^fl/fl Vav‐Cre^, and miR‐497/195^fl/fl Vav‐Cre^ mice was taken monthly (10–17 months), and the percentage of CD5^+^ B cells out of total CD19^+^B220^+^ B cells was quantified by flow cytometry. Individual measurements are connected by black lines. (B) Statistical analysis for the percentage of CD5^+^ B cells in the spleen and spleen weight of the respective mouse genotypes at an age of 17 months. Each dot represents an individual mouse (control^Vav‐Cre^, *n* = 3; miR‐15a/16‐1^fl/fl Vav‐Cre^, *n* = 5; miR‐497/195^fl/fl Vav‐Cre^, *n* = 4). (C) NK cells defined by NK1.1 expression were classified as stage I to stage IV based on their expression of CD27 versus CD11b. (D) Percentages of NK stage I to stage IV cells in control^Vav‐Cre^ (*n* = 9), miR‐15a/16‐1^fl/fl Vav‐Cre^ (*n* = 6), and miR‐497/195^fl/fl Vav‐Cre^ (*n* = 8) mice. (E) Representative FACS plot illustrating the gating of pro‐B and pre‐B (AA4.1+IgM−), and immature (AA4.1+IgM+) and mature B cells (AA4.1−) in the bone marrow. (F) Bar graphs show the percentage of pro‐ and pre‐, immature, and mature B cells within the B220^+^CD19^+^ B‐cell population (bone marrow) of control^Vav‐Cre^, miR‐15a/16‐1^fl/fl Vav‐Cre^, and miR‐497/195^fl/fl Vav‐Cre^ mice (*n* ≥ 6). Error bars depict the standard deviation of the mean. **P < 0.01; ***P < 0.001.

One key phenotype reported upon deletion of miR‐15a/16‐1 or miR‐15b/16‐2 clusters is an increase in CD5^+^ CLL‐like B cells in the spleen and blood of aged mice, indicating that deletion of one cluster can provoke a clear effect even in the presence of the two remaining clusters [8, 9]. Wondering whether loss of the miR‐497/195 cluster in mice might also recapitulate the human disease, we quantified the percentage of these cells upon Vav‐Cre‐mediated miR‐497/195 deletion and compared it to those found in miR‐15a/16‐1^fl/fl Vav‐Cre^ mice and healthy controls. Notably, whereas loss of the miR‐15a/16‐1 cluster provoked the accumulation of CD5^+^ B cells in the blood of 10‐month‐old mice and their age‐dependent increase, controls and mice lacking miR‐497/195 did not show such a phenotype (Fig. 2A). An increased percentage of CD5^+^ B cells was also detectable in the spleen, albeit less pronounced, and was accompanied by an increased spleen weight for miR‐15a/16‐1^fl/fl Vav‐Cre^ mice (Fig. 2B). However, no alterations were detectable in miR‐497/195^fl/fl Vav‐Cre^ mice, and even 17‐month‐old mice showed no signs of CLL or any other type of hematopoietic malignancy as indicated by the quantification of the major hematopoietic subsets (data not shown). Thus, unlike its paralog clusters miR‐15a/16‐1 and miR‐15b/16‐2, miR‐497/195 does not function as a tumor suppressor in the hematopoietic system.

### Normal NK and early B‐cell development in miR‐497/195^fl/fl Vav‐Cre^ mice

Beyond suppression of CLL, a recent study has reported a critical role of the miR‐15a/16‐1 cluster on splenic NK cell development [16]. Confirming these results, loss of miR‐15a/16‐1 expression upon Vav‐Cre‐mediated recombination did partially block NK cell development at the stage III to stage IV transition (Fig. 2C,D); however, deletion of the miR‐497/195 cluster had no effect compared with the control. Based on our own work, which indicated a functional role of the miR‐15 family on early B lymphocyte development [11], we furthermore assessed pro‐/pre‐B‐cell, immature B‐cell, and mature B‐cell populations in the bone marrow of miR‐15a/16‐1^fl/fl Vav‐Cre^ and miR‐497/195^fl/fl Vav‐Cre^ mice. Validating our previous *in vitro* data, deletion of miR‐15a/16‐1 indeed increased the percentage of pro‐ and pre‐B cells, indicating a developmental defect at this stage (Fig. 2E,F). Loss of miR‐497/195, in contrast, did not impair early B‐cell development. We therefore conclude that the miR‐15a/16‐1 and miR‐497/195 clusters confer different roles in immune cell development.

### **Immune cell homeostasis and function are not affected upon loss of the miR‐497**/**195 cluster**

**Fig. 3:**
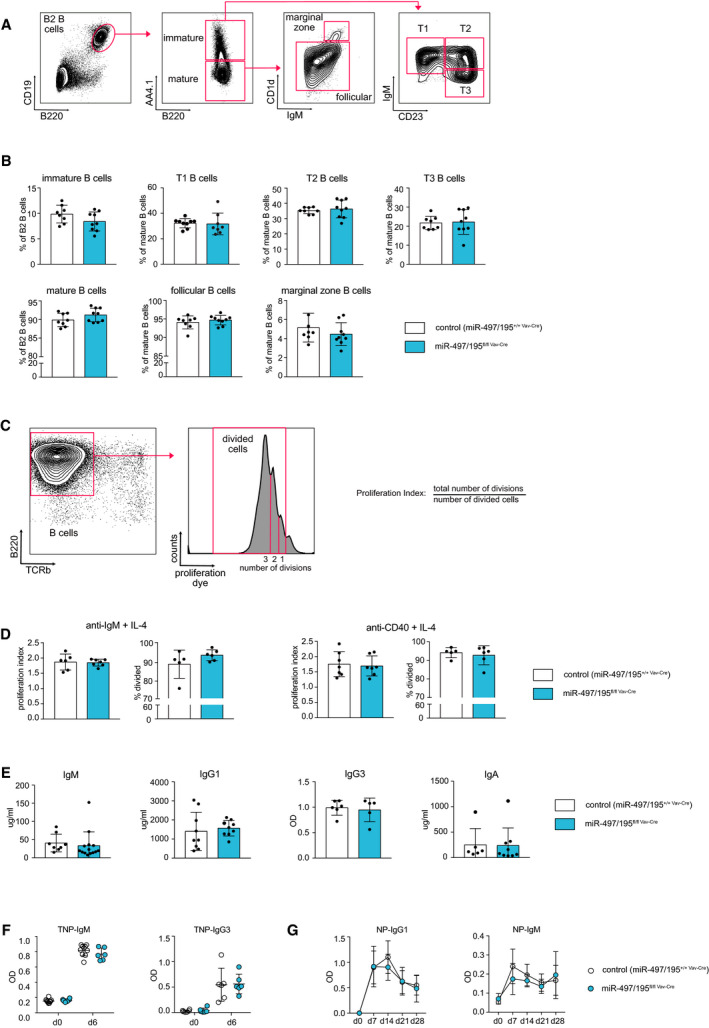
Normal splenic B‐cell development and function upon hematopoietic deletion of the miR‐497/195 cluster. (A) Gating strategy for the identification of different B‐cell subsets in the spleen. CD19^+^B220^+^ B2 B cells were divided into mature (AA4.1−) and immature B cells (AA4.1+). The mature B cells were further split into CD1d^+^ marginal zone B cells and CD1d^−^ follicular B cells. Immature B cells were gated for the different transitional phases T1 (IgM^+^CD23^−^), T2 (IgM^+^CD23^+^), and T3 (IgM^−^CD23^+^). (B) Bar graphs indicate the mean percentages of the indicated B‐cell population of miR‐497/195^+/+ Vav‐Cre^ control (*n* ≥ 7) and miR‐497/195^fl/fl Vav‐Cre^ mice (*n* ≥ 8) within the B2, mature, or immature B‐cell pool. Each dot represents the data derived from one mouse. (C, D) Splenocytes of control (*n* ≥ 5) or miR‐497/195^fl/fl Vav‐Cre^ mice (*n* ≥ 6) were labeled with a proliferation dye and stimulated with either anti‐IgM or anti‐CD40 antibodies together with IL‐4 for 72 h. The percentage of proliferated B cells (B220^+^) was quantified by flow cytometric analysis, and the proliferation index was calculated as the total number of divisions normalized to the number of divided cells (C). (E) Serum immunoglobulin levels for IgM, IgG1, and IgA were measured by ELISA and calculated according to the standard curve. The graph for IgG3 depicts the optical density (OD) as measured by the plate reader (*n* ≥ 6). (F, G) For immunizations, at least six control and miR‐497/195^fl/fl Vav‐Cre^ mice were injected with TNP‐Ficoll (F) or with NP‐CGG (G) at d0. TNP‐specific IgM or IgG3 levels as well as NP‐specific IgM or IgG1 levels were quantified at the indicated time points by ELISA. Error bars depict the standard deviation of the mean.

These data raised the question whether the miR‐497/195 cluster is implicated in immune cell development, homeostasis, and function at all. We therefore investigated whether deletion of the miR‐497/195 cluster impacts on later B‐cell development, steady‐state composition of B‐cell subsets, or immune function in general (Fig. 3). Here, transitional B‐cell subsets T1, T2, and T3 in the spleen as well as follicular and marginal zone B‐cell populations were not perturbed in miR‐497/195^fl/fl Vav‐Cre^ mice compared with controls (Fig. 3A,B). We therefore conclude that the miR‐497/195 cluster is dispensable for B‐cell development and B‐cell homeostasis and can be well‐compensated for by the other family members. To investigate whether its loss impairs B‐cell function, we furthermore isolated splenocytes of miR‐497/195^fl/fl Vav‐Cre^ and control mice and stimulated them with anti‐IgM and anti‐CD40 antibodies together with IL‐4, all of which mimic B‐cell activation during an immune response. The induced proliferation as an indicator of B‐cell activation was quantified by the percentage of divided cells and by calculation of the proliferation index (Fig. 3C). However, splenocytes from miR‐497/195^fl/fl Vav‐Cre^ and control mice did not show any differences in proliferation upon stimulation (Fig. 3D), indicating that proper B‐cell activation does not require miR‐497/195 cluster expression. Along the same line, basal IgM, IgG1, IgG2, and IgA antibody isotype levels, which allow an assessment of B‐cell development as well as B‐cell activation *in vivo*, were not perturbed in miR‐497/195^fl/fl Vav‐Cre^ mice (Fig. 3E). Supporting these data, both the miR‐497/195^fl/fl Vav‐Cre^ and control mice produced the same amount of TNP‐specific IgM and IgG3 antibodies 6 days after immunization with the T‐cell‐independent antigen TNP‐Ficoll (Fig. 3F). Likewise, miR‐497/195^fl/fl Vav‐Cre^ mice did not show any defects in their response to the T‐cell‐dependent antigen NP‐CGG over the course of 28 days, that is, they were capable to produce both high‐ and low‐affinity NP‐specific IgM and IgG1 antibodies, respectively (Fig. 3G). Together, this indicates that the expression of the miR‐497/195 cluster is not required for proper B‐cell function *in vivo*.

**Fig. 4:**
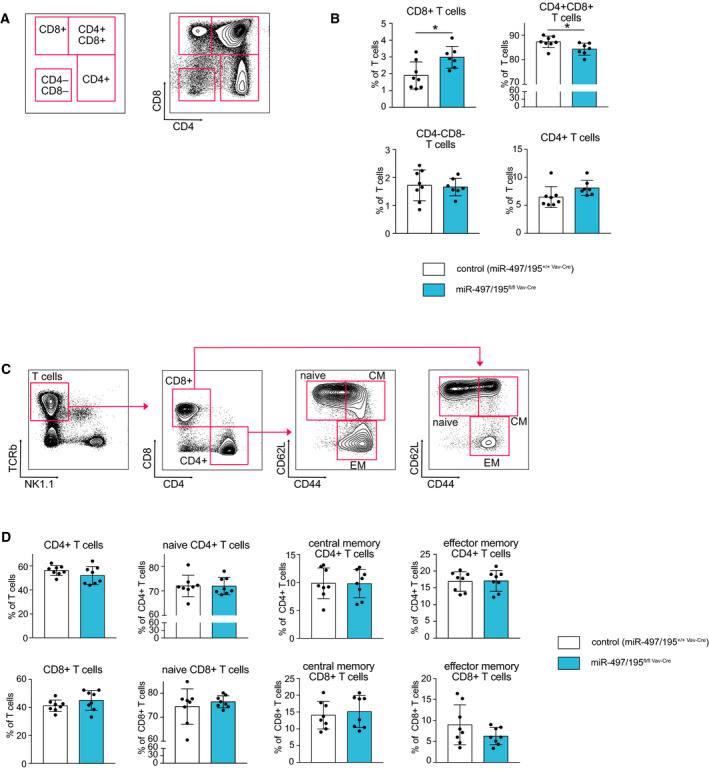
Normal T‐cell development and homeostasis upon deletion of the miR‐497/195 cluster. (A) Representative FACS plot illustrating the gating of double‐negative, double‐positive, and single‐positive CD4/CD8 T cells within the thymus. (B) Bar graphs show the percentage of developing T cells within the lin^−^ population in the thymus of miR‐497/195^+/+ Vav‐Cre^ control (*n* = 8) and miR‐497/195^fl/fl Vav‐Cre^ (*n* = 7) mice as gated in A. (C) Gating strategy for the identification of different T‐cell subsets in the spleen. TCRbeta^+^ CD4^+^ and CD8^+^ T cells were divided into naive (CD62L^+^ CD44^−^), central memory (CD62L^+^ CD44^+^), and effector memory cells (CD62L^−^ CD44^+^). (D) Bar graphs indicate the mean percentages of the indicated populations in miR‐497/195^+/+ Vav‐Cre^ control (*n* = 8) and miR‐497/195^fl/fl Vav‐Cre^ (*n* = 8) mice for CD4^+^ (upper row) and CD8^+^ (bottom row) T cells. Error bars depict the standard deviation of the mean. *P < 0.05.

Correspondingly, we also assessed a possible influence of miR‐497/195 deletion on developing and mature T cells (Fig. 4), as recent data have indicated a role of miR‐15a/16‐1 and miR‐15b/16‐2 in memory T‐cell differentiation [17]. In the thymus, miR‐497/195^fl/fl Vav‐Cre^ and control mice showed a comparable double‐negative T‐cell population, suggesting a normal initial T‐cell development (Fig. 4A,B). However, we then found a slight reduction in CD4^+^CD8^+^ double‐positive cells upon loss of miR‐497/195 cluster expression, whereas percentages of CD4^+^/CD8^+^ single‐positive T cells increased, albeit not statistically significant for the former (Fig. 4A,B). The peripheral T‐cell subsets, on the other hand, in particular central and effector memory T cells, were not affected under steady‐state conditions (Fig. 4C,D), implying that T‐cell homeostasis does not depend on miR‐497/195 cluster expression.

### Whole‐body deletion of the miR‐497/195 cluster does not result in increased tumor development

**Fig. 5:**
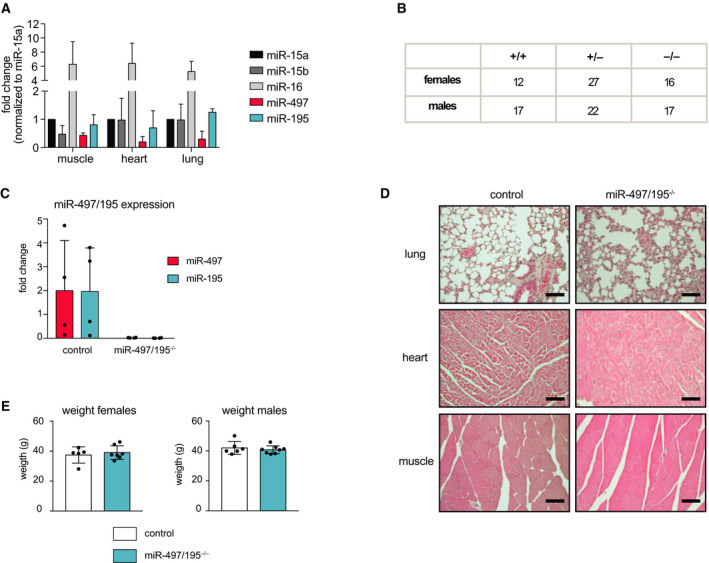
Undisturbed development and aging in whole‐body miR‐497/195^−/−^ mice. (A) miR‐497 and miR‐195 expression in the muscle, heart, and lung of three mice was measured by qPCR and compared to their paralogs miR‐15a, miR‐15b, and miR‐16. For each tissue, individual values were normalized to miR‐15a expression. (B) Table depicting the genotypes of the offspring from a cross of heterozygous miR‐497/195^+/−^ mice. (C) Quantitative PCR analysis of total RNA derived from the lungs of control (*n* = 4) or miR‐497/195^−/−^ mice (*n* = 4). (D) Histological analysis of lung, heart, and muscle tissue of control or miR‐497/195^−/−^ mice (hematoxylin and eosin, scale bars have a length of 200 µm). Shown data are representative of two animals per genotype. (E) Weight distribution of control and miR‐497/195^−/−^ mice after 17 months. Each dot corresponds to one individual mouse (control: female *n* = 5, male *n* = 6; miR‐497/195^−/−^: female *n* = 6, male *n* = 8). Error bars depict the standard deviation of the mean.

Our findings that miR‐497/195 is neither implicated in B‐cell transformation nor in B‐ and T‐cell development and function made us wonder whether this cluster may have a function outside the hematopoietic system. In fact, relative expression of the miR‐497/195 cluster in tissues such muscle, heart, and lung is substantially higher when compared to the hematopoietic system (Fig. 5A), implying functional relevance. Supporting this, numerous publications have proposed tumor‐suppressive effects of the miR‐497/195 cluster in various nonhematopoietic cancer settings [22, 23, 24, 25, 26, 27, 28]. To investigate this in more detail, we crossed miR‐497/195^fl/fl^ mice with a ubiquitous Cre deleter strain to generate miR‐497/195^−/−^ mice lacking the cluster in all tissues. Such mice were born at the expected Mendelian frequencies despite proper deletion of the cluster (Fig. 5B,C). Moreover, miR‐497/195^−/−^ knockout mice had normal body weights and showed no aberrant behavior. Their macroscopic tissue architecture and function appeared normal, and likewise, no defects were found in a detailed histological assessment of heart, muscle, and lung. Furthermore, a longitudinal analysis demonstrated normal lifespan and no signs of spontaneous tumor development over an observation period of 18 months (Fig. 5D,E and data not shown). Our findings therefore indicate that the miR‐497/195 cluster is dispensable for pre‐ and postnatal development and healthy aging.

## Discussion

Given the importance of both the miR‐15a/16‐1 and miR‐15b/16‐2 clusters in suppression of CLL on the one hand and immune cell development on the other hand, we here addressed the question whether the third paralog cluster of the miR‐15 family, miR‐497/195, confers a similar function. However, unlike miR‐15a‐16‐1^fl/fl Vav‐Cre^ mice, pan‐hematopoietic deletion of miR‐497/195 did not induce CLL‐like symptoms, such as the accumulation of CD5^+^ B cells in blood and spleen. Moreover, we failed to detect any involvement of the miR‐497/195 cluster in physiologic B and NK cell development and in lymphocyte function as determined by the response to mitogens *in vitro* and immunization with T‐independent or T‐dependent antigens *in vivo*.

In the context of CLL, the most likely explanation for the lack of a phenotype in miR‐497/195‐deficient mice is probably that the total dose of miR‐15 family members is the main determinant, rather than a specific function of one of the individual clusters. Previous data have indicated that loss of either miR‐15a/16‐1 or miR‐15b/16‐2 enables the outgrowth of CLL‐like cells [8, 9]. Given that B cells are normally characterized by equally high miR‐15a/16‐1 and miR‐15b/16‐2 expression, it is tempting to speculate that CLL can develop once the levels of miR‐15 family members fall below a certain minimal threshold. In this context, loss of miR‐497/195 may simply not provoke leukemia because its expression in the hematopoietic system in relatively low. The same probably holds true for NK and B‐cell development; however, the minimal dose of miR‐15 family members required to prevent a phenotype may vary. NK cells already show a defective development upon deletion of one cluster [16]. Along the same line, we have reported an *in vitro* B‐cell developmental delay with inhibition of all miR‐15 family members, that is, upon severe reduction in functional miR‐15 family species [11]. Supporting this, we here describe a mild developmental block at the pro‐/pre‐B‐cell stage in the absence of miR‐15a/16‐1, but not upon loss of the miR‐497/195 cluster. In the T‐cell compartment, surprisingly, hematopoietic deletion of miR‐497/195 induced an increase in thymic CD8^+^ T cells, and at least a trend toward an increase in CD4^+^ T cells, at the expense of CD4^+^/CD8^+^ double‐positive T cells. This may suggest a specific role of the miR‐497/195 cluster in T‐cell development despite its rather weak expression; however, cluster dosage also appears to play a role here, as a similar increase in CD8^+^ T cells has also been reported once both miR‐15a/16‐1 and miR‐15b/16‐2 clusters are deleted in the CD4‐Cre model [17]. This underlines the overlapping role of the miR‐15 family clusters and impedes the precise assessment of the hematopoietic role of miR‐497/195 based on its loss‐of‐function phenotype in the presence of the other miR‐15 family members. We therefore plan to combine the deletion of miR‐497/195 with that of miR‐15a/16‐1, miR‐15b/16‐2, or even both clusters. If this combined loss‐of‐function differs from deletion of only the miR‐15a/16‐1 and/or miR‐15b/16‐2 clusters, this would strongly indicate a miR‐497/195‐specific function.

One important question that emerges from these hypotheses, however, is why the miR‐15 system is built with such a high level of redundancy in the first place. In the hematopoietic context, the relatively high expression of the two main clusters supports their critical role: While loss of one cluster is tolerated at least for some time, that is, until the onset of CLL, the combined deletion of miR‐15a/16‐1 and miR‐15b/16‐2 clusters has severe consequences and provokes an aggressive form of acute myeloid leukemia early in life [29]. The miR‐497/195 cluster, on the other hand, has a different expression pattern, with higher levels in heart and lung. For us, this raised the question whether this cluster may confer a critical role beyond the hematopoietic system, either on its own, or in cooperation with other miR‐15 family members. In fact, a recent study by Yang *et al*. [18] has already demonstrated a specific role of the miR‐497/195 cluster in bone marrow endothelial cells, where it was necessary for proper blood vessel and bone formation. In our hands, however, the complete deletion of both miR‐497/195 alleles in all tissues did not provoke an overt phenotype, that is, mice had a normal life span and did not suffer from any tumor incidences. Surprisingly, even in tissues with rather strong miR‐497/195 cluster expression, such as lung, muscle, and heart, a detailed histological analysis did not reveal any alterations. This appears to contradict numerous studies that suggest a tumor‐suppressive role of miR‐497/195 in various cancer types, among them hepatocellular carcinoma, melanoma, colorectal cancer, lung cancer, or pancreatic cancer [24, 25, 26, 27, 30, 31, 32]. However, one has to keep in mind that our study evaluated spontaneous tumor development, that is, we assessed whether loss of miR‐497/195 on its own is sufficient to prime tissues for aberrant growth. However, it may very well be that miR‐497/195 inactivation promotes tumorigenesis only as a second or third hit, and that other driver mutations are necessary to reveal its tumor‐suppressive function. To test this, we plan to investigate whether loss of the miR‐497/195 cluster affects tumor onset and burden in well‐established tumor models such as KrasG12D‐driven lung cancer and diethylnitrosamine‐induced liver carcinogenesis, two cancer entities that have been associated with miR‐497/195‐mediated tumor suppression in the literature [24, 25, 30, 32].

Together, our data demonstrate differential roles of the miR‐15a/16‐1 and miR‐497/195 clusters for immune cell development and homeostasis. This may indicate that the miR‐497/195 cluster is redundant to its paralogs, that is, that its loss can be easily compensated at least under steady‐state conditions. We cannot exclude that it also possesses a unique function under certain physiological and pathological conditions; however, such conditions have to be evaluated in future projects.

## Materials and methods

### Ethics statement

Experimental procedures with animals were discussed and approved by the institutional ethics and animal welfare committees of the University of Veterinary Medicine Vienna and the Medical University of Innsbruck in accordance with good scientific practice guidelines and national legislation (license numbers: BMBWF‐68.205/0023‐II/3b/2014 and BMBWF‐66.011/0021‐V/3b/2019).

### Animals

The conditional miR‐497/195 and miR‐15a/16‐1 alleles were generated by CRISPR/Cas9‐facilitated homologous recombination in murine ES cells. In short, KH2 ES cells ([33], C57BL/6 x 129/Sv background, kindly provided by J. Zuber, IMP, Vienna) were electroporated (Nucleofector Kit; Lonza, Basel, Switzerland) with two Cas9/sgRNA vectors encoding GFP as a marker and the targeting DNA template containing the miRNA cluster flanked by loxP sites (sequences of primers for genotyping, sgRNA sequences, and the targeting template sequences are available upon request). After 36 h, ES cells were sorted for GFP^hi^ cells and plated at a low density on feeder cells. Individual ES cell clones were screened by PCR, sequenced, and then used for injection in C57BL/6NRj blastocysts. High percentage chimeras were bred with C57BL/6NRj females to confirm germline transmission and then further backcrossed to generate a congenic strain. Cre‐mediated recombination in hematopoietic stem cells was induced by mating with C57BL/6N.Cg‐Tg(Vav‐Cre) mice [21]. To induce a complete loss‐of‐function mutation, miR‐497/195^fl/fl^ mice were crossed with a CMV‐Cre strain (C57BL/6N‐Tg(CMV‐cre)1Cgn) in which the *cre* gene is under control of a human cytomegalovirus minimal promoter [34].

Animals were kept specific pathogen‐free according to FELASA recommendations [35] under controlled environmental conditions (temperature 22 °C ± 1 °C, relative humidity of 40–60%), a 12:12‐h light/dark cycle, in a facility for laboratory rodents. Food (regular mouse diet) and water were provided *ad libitum*. Mice were maintained in small groups in individually ventilated cages lined with wood shavings as bedding and enriched with nesting material. If not stated otherwise, mice were analyzed at an age of 10–12 weeks. For all experiments, male and female mice were used in comparable frequencies.

### Preparation of single‐cell suspension

Single‐cell suspensions for flow cytometry and proliferation assays were obtained by pulpifying spleens and lymph nodes through a 70‐µm filter. For bone marrow cell suspensions, femurs and tibiae were isolated, ground, and filtered through a 70‐µm filter. Lysis of erythrocytes for spleen and blood samples was performed by incubating the cells for 3–5 min in 1 mL lysis buffer (155 mm NH_4_Cl, 10 mm KHCO_3_, 0.1 mm EDTA; pH 7.5). Cells were resuspended and washed in FACS buffer (PBS with 1% FCS; Thermo Fisher Scientific, Waltham, MA, USA; 10270‐106).

### Flow cytometry

Single‐cell suspensions were stained in 96‐well plates with 30 µL of the antibody cocktails for 20 min at 4 °C. Nonspecific antibody binding was blocked by pre‐incubating the cells with anti‐CD16/31 antibodies in 30 µL FACS buffer for 10 min at 4 °C. All centrifugation steps were performed with 530 ***g*** for 2 min. For the antibody cocktails, the following antibodies were used: anti‐B220‐BV510 (BioLegend, San Diego, CA, USA; 103247, 1 : 300), anti‐CD19‐BV605 (BioLegend; 115540, 1 : 300), anti‐AA4.1‐PE/Cy7 (BioLegend; 136507, 1 : 300), anti‐AA4.1‐APC (BioLegend; 136510, 1 : 300), anti‐CD25‐PE (BioLegend; 102007, 1 : 300), anti‐cKit‐APC (BioLegend; 135108, 1 : 300), anti‐CD1d‐PE (Thermo Fisher Scientific; 12‐0011‐82, 1 : 400), anti‐CD23‐PE/Cy7 (BioLegend; 101614, 1 : 300), anti‐IgM‐FITC (BioLegend; 406506, 1 : 300), anti‐IgD‐PerCpCy5.5 (BioLegend; 405710, 1 : 300), anti‐CD5‐PE (eBioscience/Thermo Fisher Scientific, Waltham, MA, USA; 12‐0051‐83, 1 : 300), anti‐TCR‐FITC (eBioscience; 11‐5961‐85, 1 : 300), anti‐CD4‐APC‐Cy7 (BD Biosciences, San Jose, CA, USA; 552051, 1 : 300), anti‐CD8‐BV421 (BioLegend; 100753, 1 : 300), anti‐CD62L‐PE (BD Biosciences; 553151, 1 : 400), anti‐CD44‐BV510 (BD Biosciences; 563114, 1 : 300), anti‐NK1.1‐APC (BioLegend; 108709, 1 : 300), anti‐gdTCR‐PE (BioLegend; 118108, 1 : 400), anti‐streptavidin‐BV605 (BioLegend; 405229, 1 : 400), anti‐CD27‐FITC (BioLegend; 124207, 1 : 300), anti‐CD43‐PE (BD Biosciences; 7297616, 1 : 300), anti‐NK1.1‐PeCy7 (BioLegend; 108714, 1 : 300), anti‐C11b‐APC‐Cy7 (BioLegend; 101226, 1 : 300), anti‐TCRbeta‐PerCPCy5.5 (BioLegend; 109228, 1 : 300), and anti‐B220‐FITC (BioLegend; 103206, 1 : 300). The lineage cocktail to exclude non‐T cells in the thymus combined anti‐B220‐bio (eBioscience; 13‐0452‐75, 1 : 100), anti‐CD19‐bio (BioLegend; 115504, 1 : 100), anti‐Gr‐1‐bio (eBioscience; 13‐5931‐75, 1 : 100), anti‐NK1.1‐bio (BioLegend; 108704, 1 : 100), anti‐CD11b‐bio (eBioscience; 13‐0112‐75, 1 : 100), and anti‐Ter119‐bio (eBioscience; 13‐5921‐75, 1 : 100) antibodies.

### *In vitro* proliferation assay

For the *in vitro* proliferation assay, total splenocytes were stained with the eFluor 450 proliferation dye (eBioscience; 65‐0842‐90) according to the manufacturer's instructions. Prior to stimulation, 2 × 10^5^ cells per well were seeded in a 96‐well plate. Cells were stimulated with anti‐IgM (10 µg·mL^−1^) (Jackson ImmunoResearch, West Grove, PA, USA; 115‐006‐020) or anti‐CD40 (1 mg·mL^−1^) (BioLegend; 102908) antibodies together with IL‐4 (10 µg·mL^−1^) (PeproTech, Cranbury, NJ, USA; 214‐14‐20UG) for 72 h at 37 °C, 7.5% CO_2_. For flow cytometric analysis, cells were harvested and stained with anti‐B220‐FITC and anti‐TCRbeta‐APC. The proliferation index (total number of divisions normalized to the number of divided cells) was calculated using the flowjo Software (BD Life Science, Franklin Lakes, NJ, USA).

### Immunization

For immunization experiments, 10‐ to 12‐week‐old mice were injected intraperitoneally with either 200 µL TNP‐Ficoll (40 µg/mouse) or with 200 µL alum‐precipitated NP‐CGG (100 µg/mouse). For the latter, 1 mg·mL^−1^ NP‐CGG (Biosearch Technologies, Teddington, UK; N‐5055B‐5) was mixed at a 1 : 1 ratio with a freshly prepared 10% alum solution (KAl(SO4)_2_; Sigma, St. Louis, MO, USA; 31242, in PBS). The pH was adjusted to 5.5–7.0 by addition of 10 m NaOH and measured with pH indicator strips (MACHEREY‐NAGEL, Düren, Germany; 92118). The mixture was separated by centrifugation with 2500 ***g*** for 15 s, and the pellet was washed three times with PBS. The pellet was resuspended in PBS to reach the volume of the initial mixture. Blood was taken prior to immunization and at indicated time points.

### Enzyme‐linked immunosorbent assay

Serum levels of TNP‐specific IgM, TNP‐specific IgG3, NP‐specific IgM and IgG1, or basal IgM, IgG1, IgA, and IgG3 were analyzed by ELISA. 96‐well ELISA plates (Sigma; CLS3590) were coated overnight at 4 °C with 5 µg·mL^−1^ TNP‐conjugated BSA for measuring TNP‐specific antibody titers and with 50 µg·μL^−1^ NP‐BSA (Biosearch Technologies) with a ratio of 18 : 1 or 1.7 : 1 for analyzing NP‐specific antibody titers. For determining basal antibody levels, plates were coated with 2 μg·mL^−1^ Ig capture antibody (Southern Biotech, Birmingham, AL, USA; 1010‐01). After overnight incubation, plates were washed three times with wash buffer (PBS with 0.05% TWEEN‐20) and blocked with 1% BSA in PBS. After 4 h of incubation at room temperature, plates were again washed three times with wash buffer and sera were added at prior tested optimal dilutions to ensure absorbance readings in the linear range. Following overnight incubation at 4 °C, plates were washed three times and incubated with 100 µL HRP‐conjugated anti‐mouse IgM (1020‐05), IgA (1040‐05), IgG1 (1070‐059), or IgG3 (1100‐05) (all Southern Biotech, 1 : 5000 in 1% BSA in PBS) for 4 h at room temperature. Plate‐bound antibody‐HRP complexes were detected by addition of 100 µL ABTS substrate solution [(200 µL ABTS (Stock: 15 mg·mL^−1^ in a.d.), 10 mL citrate/phosphate buffer (574 mg citric acid monohydrate in 50 mL a.d.), 10 μL H_2_O_2_] for 20 min. Absorbance was measured at 405 nm using a microplate reader (Tecan Sunrise, Tecan, Männedorf, Switzerland).

### Quantitative real‐time PCR

Organs were snap‐frozen in liquid nitrogen and subsequently ground to obtain organ powder. The powder was resuspended in 1 mL TRIzol reagent (Thermo Fisher Scientific; 15596026), and total RNA was isolated according to manufacturer's instructions. RNA was reverse‐transcribed with the miRCURY LNA RT Kit (Qiagen, Hilden, Germany; 339340) followed by SYBR Green qPCR (miRCURY LNA SYBR Green PCR Kit; Qiagen; 339356).

### Statistical analysis

Values in figures depict mean ± standard deviation, and each mouse is represented as a dot. The two experimental groups were statistically analyzed by unpaired two‐tailed Student's *t*‐tests using prism 7 software (GraphPad, San Diego, CA, USA). *P*‐values < 0.05 were considered as statistically significant, and graphs were labeled according to following scheme: ***P < 0.001, **P < 0.01, and *P < 0.05.

## Conflict of interest

The authors declare no conflict of interest.

## Author contributions

SH conceptualized the data; KH, TR, MD, and LA involved in methodology; KH and MD involved in formal analysis; KH, TR, MD, and SH investigated the data; SH and KH wrote the original draft; AV, TR, and SH reviewed and edited the manuscript; KH and SH visualized the data; AV, TR, and SH acquired funding.
